# In Silico molecular docking and molecular dynamic simulation of transferrin coated Phenytoin loaded SLNs with molecular targets of epilepsy

**DOI:** 10.1371/journal.pone.0325772

**Published:** 2025-06-20

**Authors:** Ahmad Zeb, Hussain Ali, Jehan Zeb Khan, Fawad Ali Shah, Abdullah Alattar, Fawaz E. Alanazi

**Affiliations:** 1 Department of Pharmacy, Faculty of Biological Sciences, Quaid-i-Azam University, Islamabad Pakistan; 2 Department of Pharmacology and Toxicology, College of Pharmacy, Prince Sattam bin Abdulaziz University, Al-Kharj, Saudi Arabia; 3 Department of Pharmacology and Toxicology, Faculty of Pharmacy, University of Tabuk, Tabuk, Saudi Arabia; Abasyn University, Peshawar, Pakistan, PAKISTAN

## Abstract

Epilepsy is a chronic neurological disorder characterized by recurrent seizures, affecting millions of people worldwide. Phenytoin is a widely used antiepileptic drug, but its therapeutic efficacy is limited by poor brain penetration and undesirable side effects. We have investigated the drug against the selected candidate’s protein target using Insilco analysis to check the mode of action in real time system. This makes Phenytoin a promising therapeutic drug for the management of different targets involved in Epilepsy disease. Considering this, using a wide range of computer aided drug-designing approaches, high interactions with the protein targets have been inferred against drug molecule Phenytoin. Eight receptors against Phenytoin molecules showed binding interactions during molecular docking but the top four i.e. Bcl-2, BDNF, IL-1β and Caspase showed high binding affinities with docking score of 7.8 kcal/mol, 7.7. kcal/mol. 7.4 kcal/mol and 7.1 kcal/mol respectively. The compound Phenytoin interacts with several important active side residues in the active domain of all the receptors which was further validated via molecular dynamic simulations for 100 ns time intervals. Furthermore, the complexes of Phenytoin reveal very stable dynamics with average RMSD, RMSF and ROG values with stable carbon-alpha atoms confirmation at different intervals. In conclusion, these molecules are promising and require experimental validation to prove them as epilepsy inhibitors.

## 1. Introduction

Epilepsy is a chronic neurological disorder characterized by recurrent seizures, affecting millions of people worldwide [1]. Seizures are aberrant discharges of electrical activity in the brain that impair physical function. Epilepsy affects around 50 million people worldwide, with half of them having no known etiology. It has been claimed that 70% of epileptics may be seizure-free if properly evaluated and treated [2,3]. It is a brain disease associated with a persistent susceptibility to create seizures, as well as the neurobiological, cognitive, psychological, and social implications of seizures [4]. The main goal of pharmacological therapy is to reduce the frequency and severity of seizures [5].

Phenytoin, a first-line antiepileptic drug, is widely used for the management of various types of seizures. It is a non-sedative anti-epileptic drug that is derived from hydantoin. It works by inhibiting voltage-dependent membrane sodium channels, which are responsible for raising action potential [6]. It is a well-established and cost-effective conventional medicine and has a biological half-life of 15−22 hours and a prolonged period of action for approximately 24 hours [7]. In the liver, it is extensively converted into an inactive para hydroxylated form known as (4-hydroxyphenyl) −5 phenyl-hydantoin (HPPH metabolite) via the cytochrome P450 2C19 (CYP2C19) enzyme system [8]. However, its poor aqueous solubility and limited brain penetration often result in suboptimal therapeutic outcomes and adverse effects [9]. To overcome these limitations, the development of novel drug delivery systems is crucial. Solid lipid NPs (SLNs) and nanostructured lipid carriers (NLCs) are non-toxic and low-risk nanoparticulate vehicles for drug delivery, according to nanotoxicological classification system [10]. Solid lipid nanoparticles (SLNs) have emerged as a promising platform for the delivery of poorly soluble drugs, such as phenytoin, due to their ability to improve solubility, stability, and targeted delivery [11]. Furthermore, the surface modification of SLNs with targeting ligands, such as transferrin, can enhance brain delivery and improve therapeutic efficacy [12]. Transferrin is a glycoprotein that exists naturally in plasma and is utilized as a conjugating agent alongside phenytoin [13,14]. It transports ferric ions and has a function in iron metabolism. Transferrin conjugation increases the uptake of SLNs into the brain cells [13,15]. In this study, we employed a QbD approach to develop and optimize Trf-PHT-SLNs for the treatment of epilepsy. The QbD methodology allows for a systematic and risk-based approach to the development of pharmaceutical products, ensuring consistent quality and performance [16]. Based on our findings, these Trf decorated PHT-SLNs might be a potential alternative drug delivery approach for the treatment of epilepsy.

One important aspect of the pathophysiology of epilepsy is apoptosis, or programmed cell death, which is largely regulated by important proteins including Bcl-2 and Bax. Neuronal death seen in epilepsy is caused in part by the pro-apoptotic protein Bax, which is encoded by the BAX gene [17]. During apoptosis, Bax translocated to the mitochondrial membrane, causing the release of cytochrome c and the activation of caspases. Alternatively, Bcl-2, an anti-apoptotic protein, shields neurons from stress-induced apoptosis by blocking mitochondrial outer membrane permeabilization (MOMP) [18]. A mismatch between cell survival and death brought on by the deregulation of these proteins can exacerbate the hyperexcitability and neuronal damage that characterize epilepsy [19]. Neuronal survival, development, and plasticity depend on neurotrophic factors including Brain-Derived Neurotrophic Factor (BDNF) and Neurotrophin 4 (NT4). The modulation of synaptic transmission and plasticity by BDNF has been linked to changes in seizure activity and hyperexcitability, which may result in epilepsy [20]. Despite not being directly employed in the treatment of epilepsy, knowledge of NT4-specific inhibitors’ function in neurotrophic pathways is essential for creating effective treatment plans. Furthermore, epilepsy has been linked to Toll-Like Receptor 4 (TLR4), a pattern recognition receptor involved in innate immunity, by way of its involvement in neuroinflammation [21].

Pro-inflammatory cytokines can be produced because of TLR4 activation, which adds to the inflammatory milieu seen in epileptic brains. It may be therapeutically possible to reduce neuroinflammation and seizure susceptibility by targeting TLR4-mediated pathways [22]. A transcription factor called the NF-κB p50·p65 heterodimer controls the expression of genes related to inflammation and immunological responses. Since NF-κB activation in epilepsy might worsen neuroinflammation and neuronal injury, it is worth considering as a possible therapeutic target. Additionally, because it plays a part in both neurovascular remodeling and the preservation of the blood-brain barrier (BBB), VEGF has been connected to epilepsy and angiogenesis. The pathophysiology of epilepsy may be aided by VEGF dysregulation, which can damage the blood-brain barrier. VEGF inhibitors may contribute to both seizure frequency reduction and BBB integrity maintenance. These discoveries about the functions of Bax, Bcl-2, NT4, BDNF, TLR4, NF-κB, and VEGF in epilepsy highlight how critical it is to focus on these pathways in order to create successful treatment plans [23].

Other genetic regions like smooth muscle cells in the heart and airways express β1-adrenergic receptors. Airway smooth muscle cells and heart muscle cells (myocytes) are the main cell types that express these receptors [24]. The involvement of β1-adrenergic receptors (β1-ARs) in epilepsy is not well-documented, but adrenergic signaling, including β1-ARs, may influence seizure activity. The adrenergic system modulates neuronal excitability, and stress, which activates this system, can exacerbate seizures. Some animal studies and clinical observations suggest that β-adrenergic antagonists (beta-blockers) might have anticonvulsant effects, indicating a potential indirect role of β1-ARs in epilepsy. More research is needed to clarify this connection [25]. Heme Oxygenase-1 (HO-1) has been implicated in epilepsy disorders. When HO-1 breaks down heme, it produces carbon monoxide (CO), biliverdin, and free iron. These byproducts have neuroprotective and anti-inflammatory effects, which can influence neuronal excitability and reduce seizure susceptibility. Therefore, modulating HO-1 expression could be a potential therapeutic strategy for managing epilepsy [26]. The NF-κB p50·p65 heterodimer is a transcription factor that regulates genes involved in inflammation and immune responses. In epilepsy, NF-κB activation can exacerbate neuroinflammation and neuronal injury, making it a potential therapeutic target [27]. Because of its functions in maintaining the blood-brain barrier (BBB) and neurovascular remodeling, vascular endothelial growth factor (VEGF) has been associated with epilepsy. The disruption of the blood-brain barrier caused by VEGF dysregulation can result in an increase in angiogenesis and neuroinflammation, which in turn can cause epilepsy [28]. VEGF inhibitors may assist in preserving the integrity of the BBB and lowering the incidence of seizures5. These discoveries about the functions of Bax, Bcl-2, NT4, BDNF, TLR4, NF-κB, and VEGF in epilepsy highlight how critical it is to focus on these pathways to create successful treatment plans.

Numerous genes play significant roles in epilepsy within the intricate network of metabolic pathways. Their normal functioning is disrupted, leading to neurological dysregulation. Researchers employ phenytoin to undertake computational analyses of these genes by specifically inhibiting their active areas. Currently, scientists are focusing on altering these genes’ functions to restore the delicate balance between neuronal survival and programmed cell death, or apoptosis. Techniques include minute compounds that either directly or indirectly target downstream effectors in the apoptotic cascade to alter gene activity. Molecular docking studies show the atomic-level interactions between potential inhibitors and their targets, which inform drug design. Moreover, molecular dynamics simulations reveal conformational changes over time, providing insight into dynamic stability. Due to their vital role in determining the fate of neurons, these genes are intriguing therapeutic targets. As research progresses, novel inhibitors designed to regulate their activity could be the key to curing epilepsy.

## 2. Methodology

**Fig 1:**
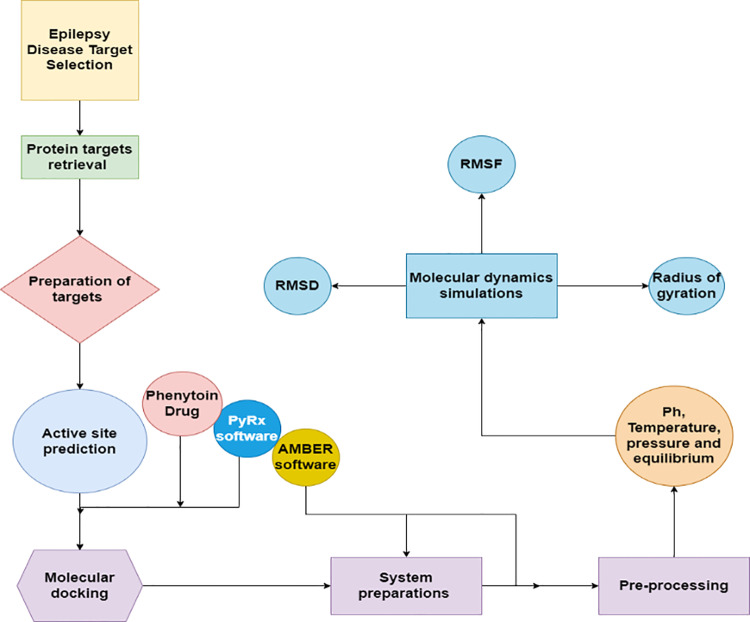
Flow chart of the current study depicting the steps used during the Insilco analysis.

The current study has been designed and investigated based on the disease, i.e., epilepsy, which is carried out during the variation in human genes. The study is divided into different sections as shown in Fig 1.

### 2.1. Inhibitor preparation

We used an FDA-approved drug chemical intended for Epilepsy disease uses for our present study. We used swissadme server [29], to check the properties known as Lipinski’s Rule [30]. Therefore, the compounds that met the requirements of Lipinski’s Rule of Five, having a molecular weight of less than 500, a logarithm of the partition coefficient (log P) ranging from −5 to 5, fewer than five donors of hydrogen bonds, and fewer than ten acceptors of hydrogen bonds—were kept for additional analysis. Furthermore, it was minimized via chimera [27] with steepest and conjugate gradient for 500 steps to make it flexible for further processing.

### 2.2. Proteins target selection

The Protein Data Bank (PDB) contains receptor targets that are pertinent to Epilepsy diseases that we have extracted. These include HO-1 (PDB ID: 1QQ8), caspase-3 (PDB ID: 1I3O), beta-1 (IL-1β) adrenergic receptor (PDB ID: 9ILB), BDNF (PDB ID: 1B98), BAX protein (PDB ID: 1F16), Bcl-2 (PDB ID: 1BXL), NDNF (PDB ID: 1B98), NF-κB p50·p65 heterodimer bound to the interferon β-κB site (PDB ID: 2I9T), the ectodomain of the human TfR (PDB ID: 1CX8), and VEGF (PDB ID: 2VPF) [31–37]. Our knowledge of these targets and how they could affect Epilepsy disorders is influenced by computational studies, such as molecular docking and dynamics simulations. Novel inhibitors that provide treatment options for Epilepsy disease management may surface as research advances.

### 2.3. Protein preparation

The protein data bank provided the high-resolution crystallographic structure of the Epilepsy disease protein targets. The Chimera AMBER ff14SB force field at UCSF was utilized to optimize the target [27]. To further enhance the structure, 750 steps of conjugate gradient were used after the 750 steepest descent steps were used to eliminate extremely detrimental steric conflicts. Eventually, minimization improves the bond geometry of the binding segments of the rigidly moving components by relaxing the structure through random search and methodical removal.

### 2.4. Molecular docking

The target protein received further preparation using AutoDockTools in PyRx prior to molecular docking [38,39]. The protein’s PDB file was changed to the Pdbqt format, water molecules and heteroatoms were extracted from the active site, and polar hydrogen atoms and charges were added using the Kollman and Gasteiger technique. Additionally, the mmff94 force field technique was utilized to optimize each medication molecule, after which they were transformed into PDBQT format. Using the AutoDock Vina virtual screening program (specifically, version 1.1.2), molecular docking analysis was performed to investigate the interaction residues between the ligands and the target protein. The active site was the aim for the grid box’s size and center points for all the target proteins. The active site coordinates for protein target BAX protein. The active site coordinates against the target proteins were obtained from their respective X-ray crystal structures which are retrieved from the protein database with literature support. Thus, further interaction study has been inferred via Discovery Studio visualizer to investigate the hydrogen bond linkages and other bonds.

### 2.5. Interaction analysis

A crucial component of molecular recognition is the directionality and contact specificity that hydrogen bonding exhibits between a protein and its ligand [40]. Thus, for quick sampling and folding kinetics, hydrogen bond energy and kinetics must be at their ideal levels. This provides stability, specificity, and protein structure needed for certain macro-molecular interactions. Thus, discovery studio was used to infer the clusters of hydrogen bond between ligand and receptor molecule [41].

### 2.6. Molecular dynamic simulations

Since proteins’ activity inside cells is dynamic, further validation of the compounds’ binding stability to the active pocket is essential to understanding dynamics. Using the AMBER16 simulation program, the simulation process was carried out. Tleap was used to record topologies, while the Antechamber software was used to produce complicated preprocessing and parameters. Using FF14SB [42] and GAFF [43], respectively, the candidates protein and compounds were treated. The systems were solvated in a 12-inch-by-12-inch water box made using the TIP3P water solvent model [44]. To produce neutral systems, counterions were also introduced at the same time. Systems were then employed for 2000 steps of the energy reduction phase. The systems’ coordinates were maintained as they were progressively heated to 310 K. By employing a constant normal temperature and pressure (NPT) ensemble for 500 ps, each system was brought to equilibrium. A 100 ns simulation production run using an NPT ensemble was conducted. To ensure that the results could be repeated, the production run was done twice, with a different starting velocity for each copy. In this process, long-range electrostatic interactions were handled using the particle-mesh Ewald procedure, with an 8 Å cut-off distance for non-bounded interactions [45]. Langevin dynamics [46] was taken into consideration to get the control temperature throughout the simulation, and the SHAKE method [47] was employed to restrict covalent bonds with hydrogen atoms. Plots were created using the XMGRACE program [48], and the simulated trajectories were examined using the CPPTRAJ module of AMBER16 [49].

## 3. Results

### 3.1. Inhibitor selection and preparation for molecular docking

Results suggest the distinct anticipated pharmacokinetic characteristics of the chemical phenytoin. Compounds with chemical name C15H12N2O2 having molecular weight of 252.27 g/mol may be excellent choices for oral delivery because of their reduced size. Additionally, it is possible that this will help the molecule absorb more readily from the gut and reach the target region at a higher concentration (Veber et al., 2002) as shown In Table 1. Additionally, supporting efficient drug absorption, this compound is somewhat soluble in water. According to Whitty’s (2011), this compound exclusively interacts with one target within the host cell and does not trigger any false positive findings due to the lack of Pan-assay interference compounds (PAINS) warnings. Furthermore, readily manufactured in the lab for experimental testing, the substances have an excellent synthetic accessibility score. It is also non-toxic for chemicals.

**Table 1:** Drug likeness and pharmacokinetics of the selected compound phenytoin.

Formula	C15H12N2O2
MW	252.27
#Heavy atoms	19
#Rotatable bonds	2
#Rotatable bonds	2
#Aromatic heavy atoms	12
Fraction Csp3	0.07
#H-bond acceptors	2
#H-bond donors	2
MR	77.5
TPSA	58.2
iLOGP	1.65
XLOGP3	2.47
WLOGP	0.9
MLOGP	1.61
Silicos-IT Log P	2.42
Consensus Log P	1.81
ESOL Log S	−3.3
ESOL Solubility (mg/ml)	1.28E-01
ESOL Solubility (mol/l)	5.06E-04
ESOL Class	Soluble
Ali Log S	−3.34
Ali Solubility (mg/ml)	1.16E-01
Ali Solubility (mol/l)	4.61E-04
Ali Class	Soluble
Silicos-IT LogSw	−5.62
Silicos-IT Solubility (mg/ml)	6.07E-04
Silicos-IT Solubility (mol/l)	2.40E-06
Silicos-IT class	Moderately soluble
GI absorption	High
BBB permeant	Yes
Pgp substrate	No
CYP1A2 inhibitor	Yes
Lipinski #violations	0
Ghose #violations	0
Veber #violations	0
Egan #violations	0
Muegge #violations	0
Bioavailability Score	0.55
PAINS #alerts	0
Brenk #alerts	1
Leadlikeness #violations	0
Synthetic Accessibility	1.83

### 3.2. Molecular docking

Virtual screening is a high throughput screening method that involves screening pharmacological compounds against a particular biological target. Finding the best receptor binders that can influence the target, sometimes known as “hits” or “leads,” is a financially advantageous method [50]. There is greater potential that new chemical scaffolds, such anti-Epilepsy chemicals, may be discovered. We have discovered the binding affinity of compound Phenytoin in complex with several targets that are involved in epilepsy metabolic pathways. Table 2 shows the distribution of this chemical based on the binding energy range in complex with target receptors. After docking experiment, it was discovered that four receptors showed best binding affinity against the Phenytoin compound. The top score of Phenytoin resulted with receptor Interleukin 1β (IL-1β) a higher binding energy of −7.8 kcal/mol. This has been followed by receptor BDNF with binding energies of −7.7 kcal/mol. Among the other receptors that bound to Phenytoin compounds had binding energies of −7.4 kcal/mol against Caspase-3 and −7.1 kcal/mol against Bcl-2. The energies were also investigated against other targets resulted in −6.9 kcal/mol against HO-1, −6.6 kcal/mol against NFkB, −6.3 kcal/mol against TNF-alpha, −6.3 kcal/mol against VEGF and the protein receptor were inferred with a binding energy of −6.6 kcal/mol using cluspro software.

**Table 2:** The bioactive chemical compound in complex with target proteins along with the docking analysis results.

Docked complex	Interactive residues	Distance (Å)	Binding affinities (kcal/mol)
Bcl2_Phenytoin complex	Arg142	1.5 Å	−7.8
BDNF_Phenytoin complex	Cys17, Val16, Cys111	2.0 Å, 1.3 Å and 2.6	−7.7
IL-1β _Phenytoin complex	His286	2.1Å	−7.4
Caspase-3_Phenytoin complex	Arg207, Ser209	1.3 Å and 2.5 Å	−7.1
HO-1_Phenytoin complex	Lys148	4.5 Å	−6.9
NFkB_Phenytoin complex	Tyr36, His88, Asn155	5.6 Å, 5.55 Å and 3.58 Å	−6.6
TNF-alpha_Phenytoin complex	Leu120	5.4 Å	−6.3
VEGF_Phenytoin complex	Tyr25	3.93 Å	−5.6
Transferrin-receptor complex	Gly609, Phe622, Cys620, Phe619, Brg602, His606, Leu607, Lsy545	3.73 Å. 3.09 Å, 3.20 Å, 2.25 Å, 3.08 Å, 3.50 Å, 3.55 Å, 4.9 Å	−6.6

### 3.3. Lead molecule binding mode analysis

**Fig 2:**
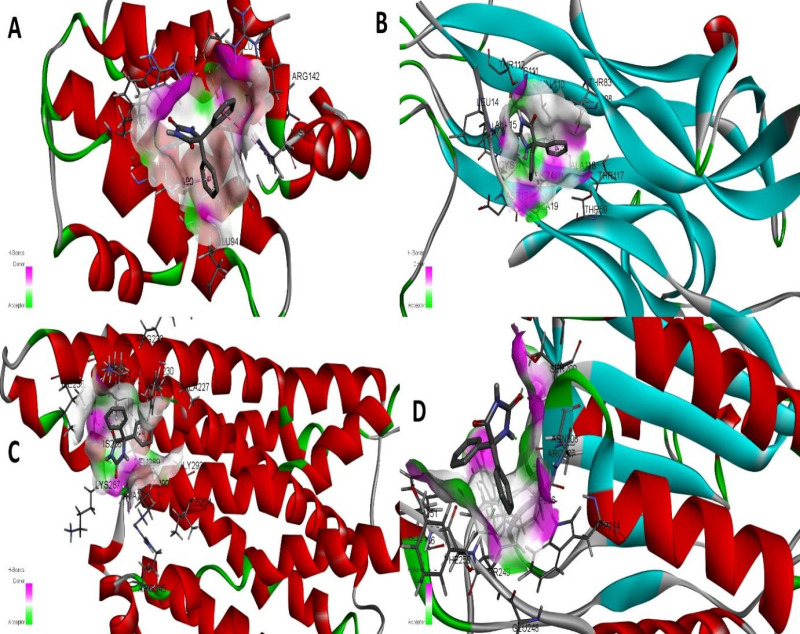
Active Binding domains of Top complex for Epilepsy disease targets with clouds of hydrogen bonds acceptor and donor atoms. (A) depicts phenytoin in complex with Bcl2 (B) shows the phenytoin in complex with BDNF (C) shows phenytoin in complex with beta1 and (D) illustrate the phenytoin in complex with caspase target along the key interacting residues.

**Fig 3:**
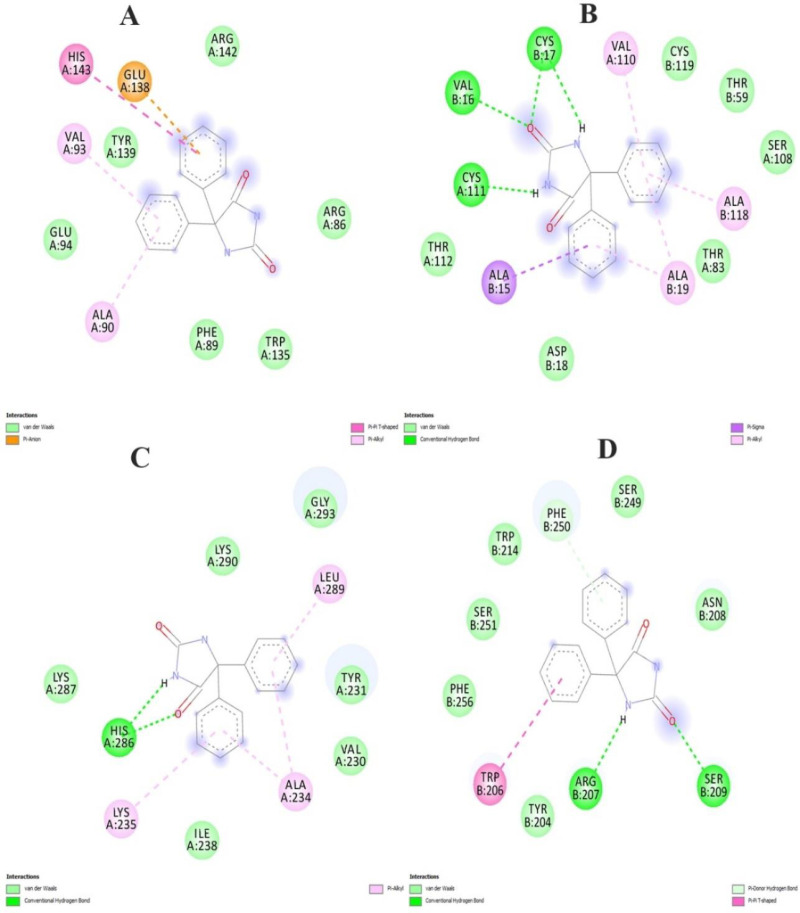
The top complexes regulate the binding shape and how the ligand molecule in complex with receptor interacts with them as shown here in this Fig. (A) BCL2 (2) BDNF (C) Beta-1 and (D) Caspase docked complex.

**Fig 4:**
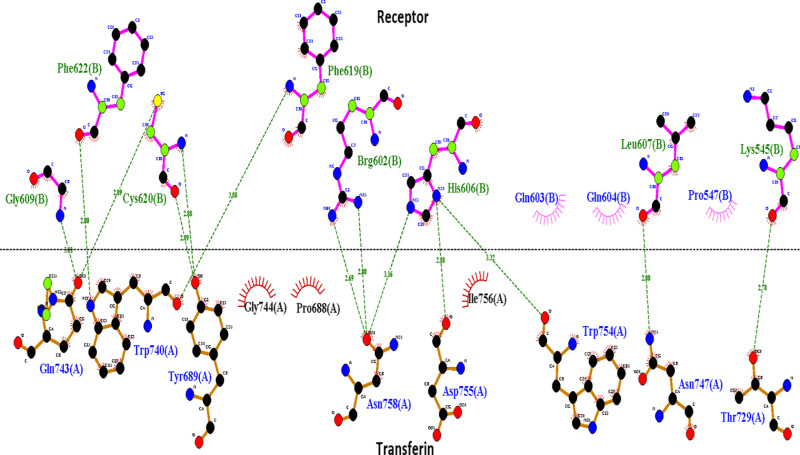
Transferin-receptor complex with binding residues involved in hydrogen and hydrophobic reactions.

The docked Transferrin receptor complex and selected Phenytoin compounds underwent further binding mode analysis to interpret the compound Phenytoin binding to the top candidate protein. To ascertain if the intermolecular docked configuration is stable enough for long-term binding and functional inhibition of all the enzymes, it is crucial to comprehend the binding conformation of Phenytoin. The enzyme’s active pocket is encircled by the critical residues Arg142 with 1.5 Å distance. This residue is strongly interacting with the moieties of compound 5,5-diphenylimidazolidine-2,4-dione showing strong hydrogen bond acceptor and donor cloud as shown in Fig 2(A). This moiety specifically forms van der Waals connections with Arg142, Tyr139 Glu94, Phe89, Arg86 and Trp135 as shown in Fig 3. In addition, several pi-alkyl and alkly-alkyl interactions have been inferred. Furthermore, another Top receptor complex (BDNF) has been investigated, resulting in the formation of Three hydrogen bonds by Cys17, Val16, Cys111 at distances of 2.0 Å, 1.3 Å and 2.6 Å Fig 3B, respectively with clouds of hydrogen bonds donor and acceptor have been depicted in Fig 2B. Similarly, we investigated the top3 complex (Beta-1) which forms hydrogen bonds with His286 at (distance 2.1Å) Fig 2C with two atom oxygen and hydrogen of the compound moiety as shown in Fig 3C, contribute significantly to maintaining the compound’s stability at the docked site. Gaining a firm root at the active pocket is the responsibility of this core moiety. Thus, we have checked for another best scoring complex system Caspas-3 which shows strong hydrogen bonds at the terminal site with Arg207, Ser209 at distance of 1.3 Å and 2.5 Å respectively contributed favorably to both interaction stability and the overall docked conformation as shown in Fig 2D and Fig 3D. Other weak hydrophobic connections and a robust van der Waals network was formed by the remaining compound structure. Finally, a receptor molecule at normal reaction during metabolic pathways were investigated for interactive analysis which leads to a strong and robust binding response with hydrophobic and hydrogen bonds formation for Transferin-receptor complex as shown in Fig 4.

### 3.4. MD simulations

**Fig 5:**
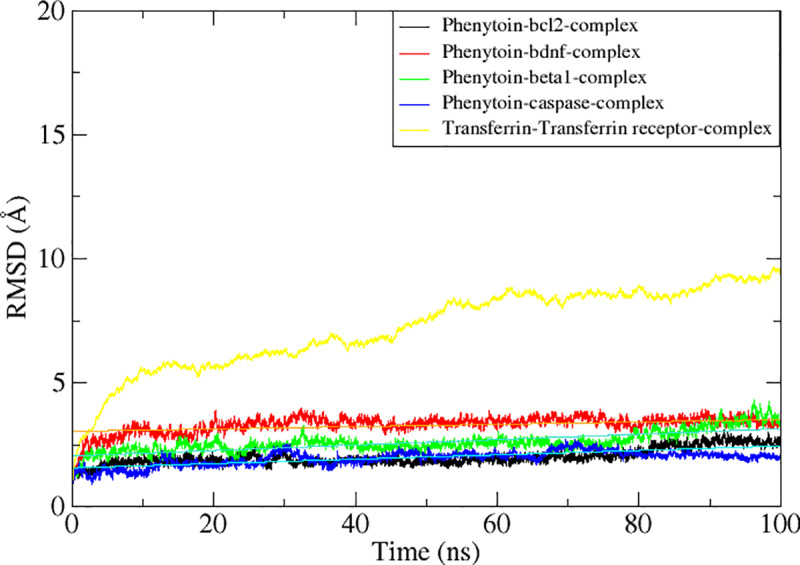
Molecular dynamics simulations-based statistical analysis (RMSD) to evaluate the dynamics and intermolecular stability of the Epilepsy target protein in complex with Phenytoin compound for 100ns simulation time intervals.

To confirm the binding affinity and conformation stability of drugs, further investigation of the docked complexes is necessary as the single docked snapshot produced by docking is sometimes unreliable. Understanding the docked complex dynamics is crucial because biomolecules exhibit dynamic behavior within cells [51]. It was discovered that the plots that were produced were identical, and the complexes’ dynamic activity could be understood. Statistical measures like root mean square fluctuation (RMSF) and root mean square deviation (RMSD) helped to reveal the intricate dynamics. Over the course of the simulation, these two parameters were produced based on the carbon alpha atoms for all the five protein complex systems. Higher conformation stability is indicated by lower RMSD, and vice versa. Fig 5 shows the RMSD plot for each complex. All the complexes investigated were quite stable, as shown in Fig 5, and there were no obvious substantial departures from the plots. As the simulation ends, the compound combined with Bcl-2 shows mean RMSD value of 2.06 Å with 3.1Å with maximum RMSD and acquire stable intermolecular conformation and dynamics as shown in Fig 5. This is followed by complex BDNF which inferred a mean value of 3.3Å with maximum value of 3.9 Å at 20 ns and then stable up to 100 ns time intervals. The top third complex beta-1 in complex with Phenytoin depicted a mean square of 3.3 Å and maximum value of 4.6 Å at 98 ns. Results showed high stability throughout the simulation intervals with confirmational changes noticed among different trajectories. Whereas a stable system has been statistically determined for complex Caspase-3 results in RMSD mean value of 1.9 Å with maximum value of 2.7 Å. Deviations at certain intervals were monitored but it does not affect the confirmation and ligand stability throughout the 100 ns time intervals as shown in Fig 5. Finally, a transferrin receptor normal behavior was monitored for 100 ns time intervals which showed a high RMSD value with mean score of 7.4 Å with maximum value of 10.4 Å. Some confirmational changes were measured during the trajectories analysis but the system remains static at most of the time interval. Thus, these simulations investigate the current trend of stability for the drug we have selected during the study against the receptor molecules which deduced a strong stable mode of action inside the active pocket of the macromolecules.

**Fig 6:**
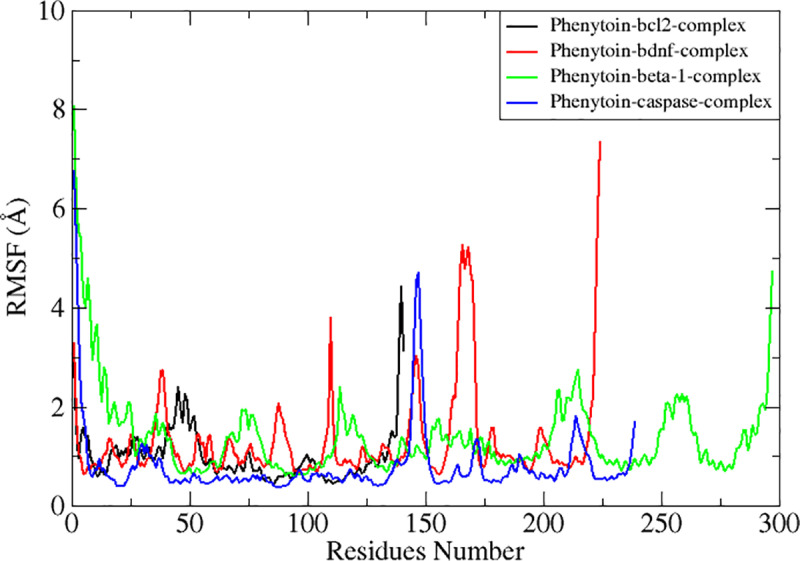
Molecular dynamics simulations-based statistical analysis (RMSF) to evaluate the dynamics and intermolecular stability of the Epilepsy target protein in complex with Phenytoin compound for 100ns simulation time intervals.

**Fig 7:**
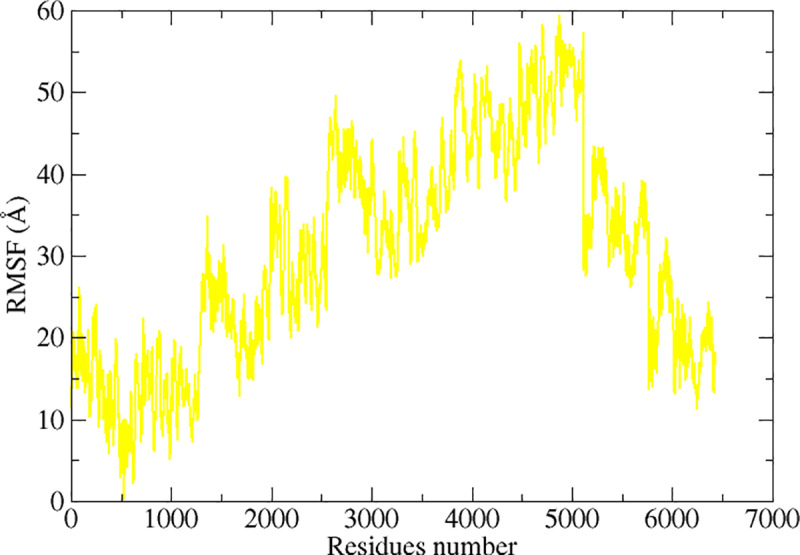
Molecular dynamics simulations-based statistical analysis (RMSF) to evaluate the dynamics and intermolecular stability of the Epilepsy target protein in complex with receptor Transferrin for 100ns simulation time intervals.

RMSF was used to compute the residue level deviation (Fig 6). The bulk of the complex’s residues were shown by the RMSF to be well within the stable deviation region, as shown by the RMSD graphs. Comparing the N-terminal domain to the C-terminal, the former was far more flexible. The RMSF values of the active residues for Bcl-2 complex, Arg142, Tyr139 and Glu94 are 1.5 Å, 0.5 Å and 3 Å respectively with average value of 1.02 Å and maximum value is 4.4 Å. This is followed by complex BDNF having an RMSF value against active residues, Cys17, Val16, Cys111 are 1.3 Å, 0.9 Å and 1.8 Å respectively with average value of 1.3 Å and maximum value is 7.3 Å. This illustrates how extremely stable these residues are and how they promote complex interactions throughout simulation. The simulated complexes’ root mean square fluctuations (RMSF) were then computed for beta-1 complex system with an average MRSF score of 1.3 Å with maximum value of 8 Å. The active residues involved in stability have shown the RMSF value ranges between 3 Å. Finding the targeted proteins’ flexible residues and comprehending how these variations impact complex stability are made easier with the use of RMSF analysis. Finally, the graphs reveal the complex caspas-3 with RMSF mean value 0.8 Å with maximum value of 6.7 Å as shown in Fig 6, only with slight variations. The transferrin receptor RMSF value has been recorded with mean value of 30 Å having maximum value of 59.4 Å. A lot of fluctuations have been recorded during simulation at different residues, but hydrogen bonds were strongly held throughout the simulation time intervals as shown in Fig 7.

**Fig 8:**
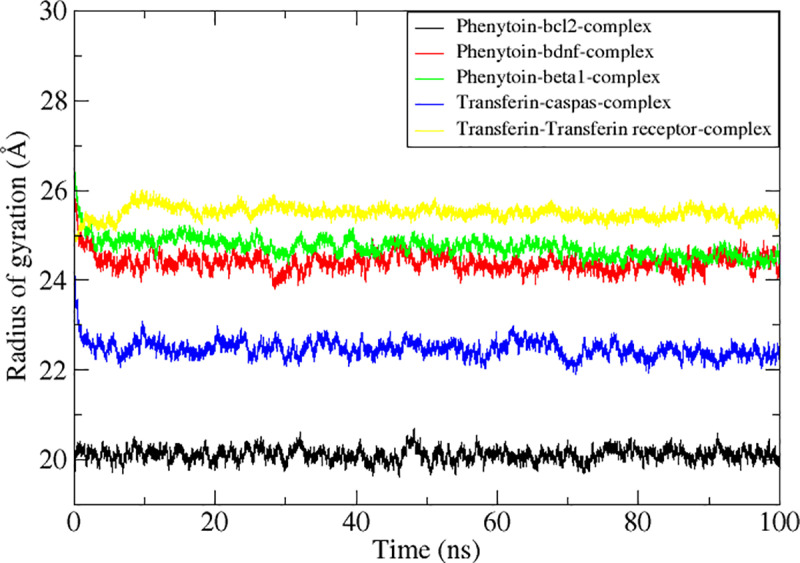
Depicting Molecular dynamics simulations-based statistical analysis (RMSF) to evaluate the dynamics and intermolecular stability of the Epilepsy target protein in complex with receptor Transferrin for 100ns simulation time intervals.

**Fig 9:**
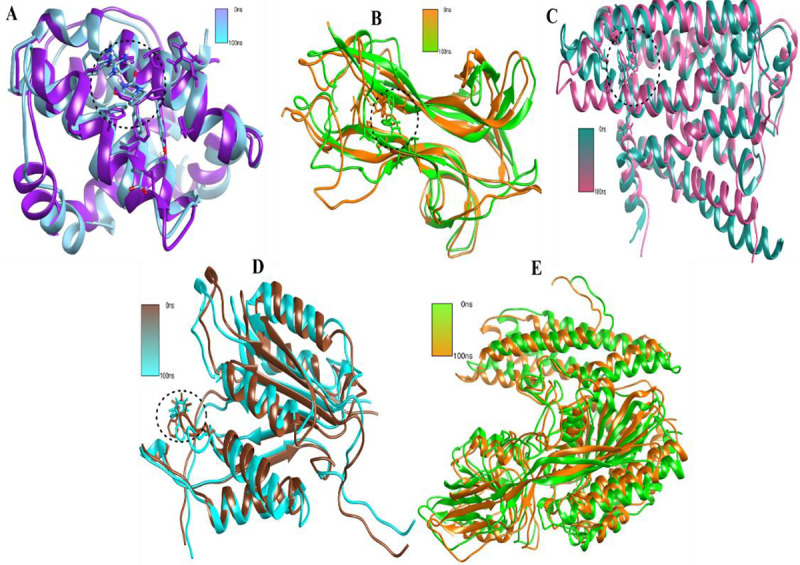
(A) Trajectories analysis of Bcl-2 complex system with initial and final frame. (B) shows the Bdnf-complex system with initial and final frame trajectory (C) depicts the beta1-complex system with initial and final frame trajectory (D) illustrates the caspase-complex system with initial frame trajectory and (E) presenting the control complex system with initial frame trajectory.

The radius of gyration (Rg), which ultimately defines the folding rate and protein stability, reveals how compact the system is. The Rg technique was utilized to ascertain the compactness, behaviors, and stability of the complexes. The results show that following the first little oscillations, the Rg of each system and the were stabilized and in agreement with the RMSD values. These findings confirmed that the complexes stayed compact and stable for the whole 100 ns simulation time (Fig. 8). A system that has a lower RoG rating is more stable and is very compact. As seen in Fig 7, the RoG values for the target Bcl-2 demonstrated good stability for over the course of 100 ns. According to all these tests, all the targeted proteins complexes showed high dynamical stability when the virtually screened compounds were present. The optimum fitting between the proteins and the chemicals, which resulted in strong association and, eventually, stable complex formation, was also proven by the complex intermolecular stability. The 2^nd^ complex BDNF also exhibited high stability in the RoG plot trajectories, while the third complex beta-1 displayed slight variations as seen in Fig 7. Followed by complex caspas-3 and the transferrin receptor complex that showed a slight variation with stable confirmatory behavior as shown in Fig 8. Finally, we have inferred confirmatory changes among all the complexes. Results deduced insights into initial and final frames for the complexes which have static confirmatory behavior for the time duration of 100 ns simulation. The superimposed frames result in a slight deviation at some point but the inhibitors remains high stability as shown in Fig 9.

## 4. Discussion

The neurological condition known as epilepsy is recurrent and is brought on by aberrant electrical activity in the brain. The disease has been responsible for around 50 new cases per 100,000 people annually, with symptoms including altered movement, bruises, and loss of awareness. There are several drugs available to treat epilepsy since the biology of the condition is not well understood. The process by which a properly functioning brain experiences changes that result in the development of epilepsy is known as epileptogenesis, and it involves a number of components [52,53]. Elevated proinflammatory cytokines and persistent inflammation in the epilepsy environment can aid in the onset and development of the condition. The hallmarks of epilepsy, neuronal hyperexcitability and seizure activity, can be brought on by inflammatory processes in the brain [54]. There are many different drugs available to treat epilepsy as the pathophysiology behind the condition is still not fully understood. There are several unique pharmacological medicines available to address this situation. Gray matter in the cortex or subcortex is where epilepsy begins, which is characterized by epileptic episodes. A sudden imbalance between excitation and inhibition within the cortical neuron network causes epileptic seizures. It begins when neurons show high levels of persistent, synchronized, and excitable neural activity [55].

We have obtained relevant receptor targets for epilepsy from the Protein Data Bank (PDB) for our investigation. These consist of the following: HO-1 (PDB ID: 1QQ8), BDNF (PDB ID: 1B98), beta-1 adrenergic receptor (PDB ID: 9ILB), BAX protein (PDB ID: 1F16), Bcl-2 (PDB ID: 1BXL), BDNF (PDB ID: 1B98), NF-κB p50·p65 heterodimer bound to the interferon β-κB site (PDB ID: 2I9T), the ectodomain of the human TfR (PDB ID: 1CX8), and VEGF dimer (PDB ID: 2VPF). These genes are linked to epilepsy and are essential for many cellular functions. A pro-apoptotic protein implicated in programmed cell death is called BAX. An increase in cell death or a decrease in apoptosis might result from BAX dysregulation, which can aggravate epilepsy. The anti-apoptotic protein Bcl-2 stops the permeabilization of the outer membrane of mitochondria. Bcl-2 dysregulation is seen in several illnesses, such as lymphomas and epilepsy. Although inhibitors do not directly target BDNF, which is essential for neuronal survival and growth, knowledge of its structure helps guide treatment strategies. Asthma and cardiovascular disorders are associated with autoantibodies that target beta-1 adrenergic receptors, which are found in heart and airway smooth muscle cells. As a result of heme breakdown, HO-1 produces free iron, biliverdin, and carbon monoxide, which have neuroprotective and anti-inflammatory effects that can influence neuronal excitability and reduce seizure susceptibility. The NF-κB p50·p65 heterodimer regulates genes involved in inflammation and immune responses, and its activation can exacerbate neuroinflammation and neuronal injury in epilepsy. VEGF is linked to epilepsy due to its roles in neurovascular remodeling and maintaining the blood-brain barrier (BBB). Dysregulation of VEGF can lead to BBB breakdown, contributing to the development of epilepsy by promoting excessive angiogenesis and neuroinflammation.

Using molecular docking and dynamics (MD) simulation, the complexes’ stability and energetic mobility were investigated. These included the radius of gyration (RG), root mean square fluctuation (RMSF), and root mean square deviation (RMSD) of the alpha carbon atoms in the complexes. The stability of compound 4 complexes and a receptor transferrin complex was observed for 100 ns, as evidenced by Fig 6, where the most active complexes had good specificity, with the RMSD values of both five complex system and the protein backbone remaining below 2 Å. Throughout the simulation, the RMSD profile of these complexes remained consistent. Our Compound demonstrated superior stability and retention inside the protein’s binding site when tested against the Bcl-2 protein, BDNF, Beta-1 Caspase and transferrin as seen by its lowest RMSD value. The RG of complexes was also calculated to evaluate receptor stability in more detail (Fig 8). RG values for the receptor were stable for the complexes and were indicated by average RG values of 20.1, 24.4, 24.7, 22.4 and 25.4 Å, respectively (Fig 8).

Molecular docking and dynamics simulations, among other computational studies, contribute to our comprehension of these targets and their possible influence on Epilepsy disorders. Thus, novel inhibitors that provide therapeutic opportunities for the management of Epilepsy disorders may surface as research advances.

## 5. Conclusions

Numerous biological processes and molecular activities like epilepsy were found by our systematic and thorough investigations of disease-gene-target-drug interaction networks. Most of these connections were linked to neuroactive ligand-receptor interactions. These findings may contribute to the creation of innovative pharmaceutical approaches and offer a fresh theoretical foundation and point of reference for the clinical management of concomitant epilepsy. This important discovery proved the benefit of employing MD simulation following docked complexes with top4 and transferrin receptor. In addition to revealing order-binding residues and altering the other residues in the catalytic site found by docking, the ligand-receptor complex from the molecular dynamics simulation also showed stable environment for the selected candidates. Further in vitro and in vivo research is required to ascertain whether compound 4 might be a drug candidate to treat Epilepsy illnesses. This work might serve as an example of an in-silico approach to finding novel anti-Epilepsy disease inhibitors.
